# Atypical Wells syndrome successfully treated with dupilumab

**DOI:** 10.1002/ski2.206

**Published:** 2023-02-24

**Authors:** Patrick McMullan, Kristin Torre, Sueheidi Santiago, Gillian Weston, Jun Lu

**Affiliations:** ^1^ Department of Dermatology University of Connecticut Health Center Farmington Connecticut USA

## Abstract

Wells syndrome, also referred to as eosinophilic cellulitis, is a rare and often sporadic inflammatory skin condition whose aetiology remains uncertain. Clinically, this condition presents as a collection of erythematous, oedematous, and tender skin lesions most often affecting the extremities and trunk that can mimic cellulitis. Histologically, Wells syndrome is characterised by inflammatory changes and eosinophilic infiltration of the dermis with the absence of underlying infection, thereby distinguishing it from cellulitis. Due to the rarity of this syndrome and its ambiguous presentation, there remains to be a definitive strategy for treatment. Recent case reports have documented varying success and recurrence with the use of oral and topical corticosteroids, antifungals, antibiotics, immunosuppressants and antihistamines. Here, we report a unique case of progressively worsening neutrophilic‐rich Wells syndrome on the vertex of the scalp that was successfully treated with a combination of dupilumab and oral corticosteroids.

## Graphical abstract

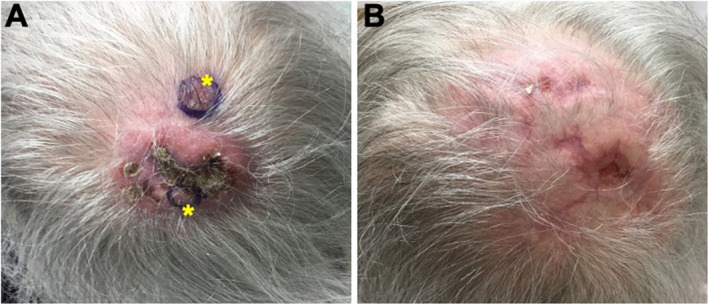

## 1 CASE REPORT

An 85‐year‐old Caucasian female presented to the dermatology clinic with a 2.5‐month history of a gradually enlarging mass on the vertex of the scalp. The patient was initially seen by her primary care physician for a nontender plaque with occasional clear drainage. However, the lesion continued to expand despite completing courses of griseofulvin (500 mg daily for 30 days) and Cephalexin (500 mg three times daily for 10 days), leading to a dermatology referral. Additional work‐up included a head CT without IV contrast which showed a homogenous area of soft tissue thickening in the scalp without underlying bone or dural involvement. She denied any recent travel, trauma, changes in medication, or history of skin cancer and review of systems was negative for fever, chills, and weight loss.

**FIGURE 1:**
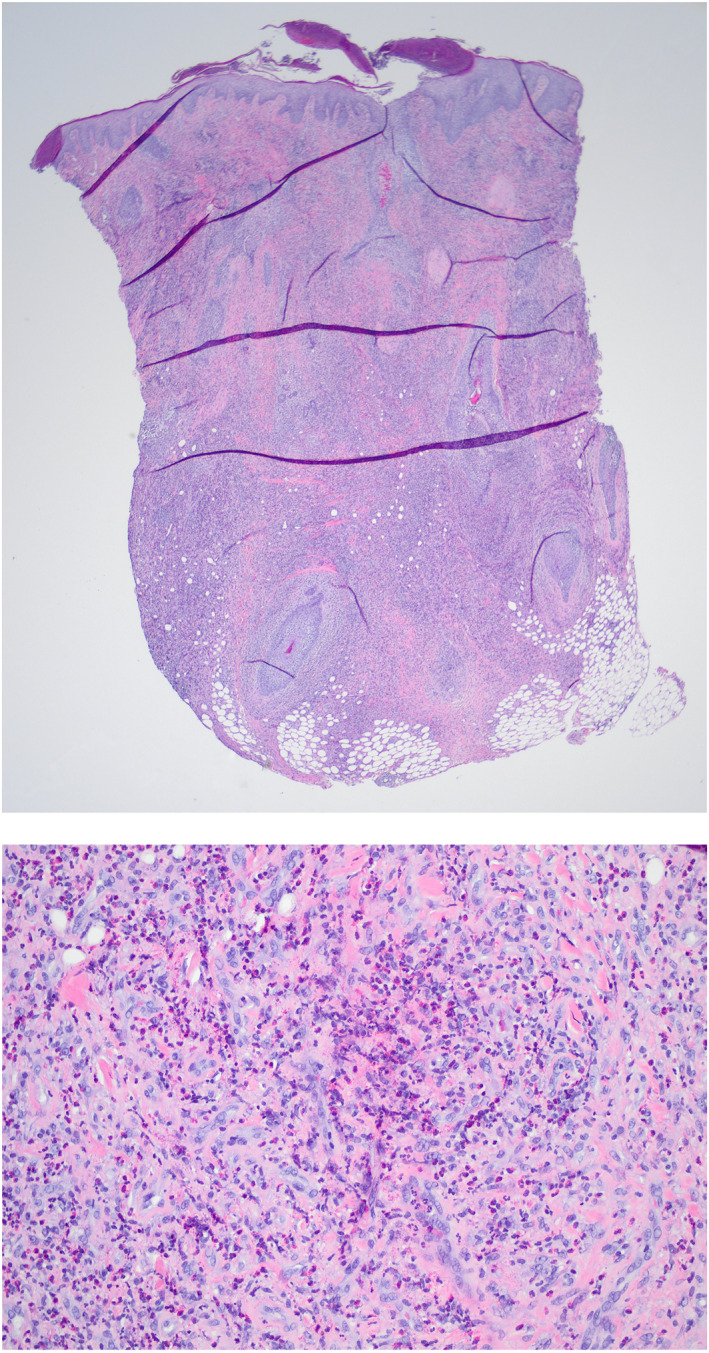
Representative images of low (20×) and high‐power (200× and 400×) Haematoxylin and Eosin (H&E) stained punch biopsy obtained from occipital scalp demonstrating evidence of dermal inflammation with predominate eosinophilic with neutrophilic infiltration and scattered reactive vasculature.

On examination of the skin, there were multiple violaceous, indurated, nontender papules coalescing into a plaque with clustered nodules and central crusting on the occipital scalp. Haematoxylin and Eosin (H&E) stains demonstrated dense inflammation with predominate eosinophils and some neutrophils in higher manifestations (Figure 1). There is secondary reactive fibroblast/fibrohistocystic features and vascular ectasias but no vasculitis. Periodic acid‐Schiff (PAS), Grocott methenamine silver (GMS), and Gram and acid‐fast stains were negative. There were also no histologic features of malignancy.

**FIGURE 2:**
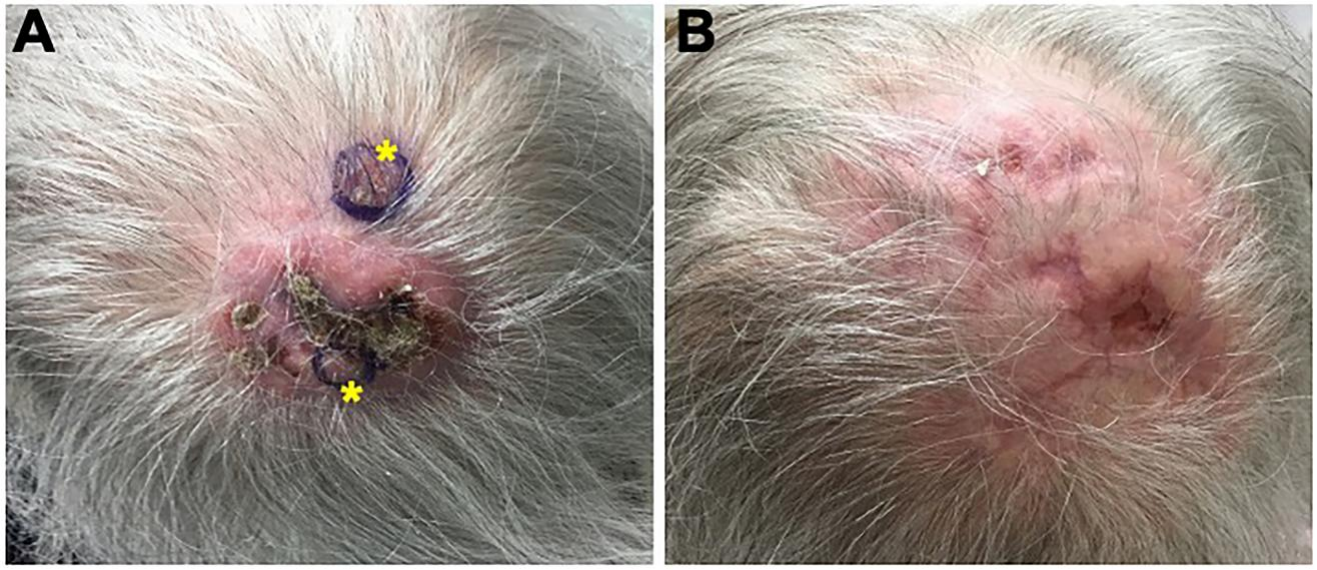
Serial clinical photographs of occipital scalp (a) before and (b) following dupilumab and oral corticosteroid treatment. The location of the two 6 mm punch biopsies are depicted by the asterisks.

When patient returned to clinic 2 weeks later, she developed new indurated, tender nodule located next to the initial growth (Figure 2a). Repeat biopsies of the left vertex and midline scalp were taken for both routine histology and tissue culture. Pathology confirmed the presence of diffuse, focally dense eosinophilic inflammatory infiltrate with associated neutrophils. Focal eosinophilic degranulation was seen with scattered reactive vessels lined by CD31+ endothelial cells. Tissue culture was negative for bacteria, fungal and mycobacteria infection. In addition, complete blood count showing leucocytosis and mild transient eosinophilia with WBC at 12.9 and eosinophils at 7.6 × 10^3^/μL respectively. Based on clinical and pathological correlation, a diagnosis of neutrophilic‐rich Wells syndrome was made.

Initial treatment with intralesional and oral corticosteroid with minocycline only lead to partial transient improvement followed by rapid relapse and rebound with larger plaque and nodules. Therefore, subcutaneous dupilumab (initial 600 mg dose followed by subsequent 300 mg every 2 weeks) was initiated in addition to oral prednisone for further management.

One month after combination therapy, the patient reported significant improvement with complete resolution of active inflammation with only residual mild erythema and post‐inflammatory hyperpigmentation (Figure 2b). Therefore, prednisone was tapered off completely and patient continues on 300 mg subcutaneous dupilumab injection every 2 weeks without re‐occurrence. She had no reported side effects with dupilumab.

## 2 DISCUSSION

Our case highlights the importance of clinicopathological correlation for diagnosing atypical presentations of Wells syndrome. Moreover, our case highlights a potential novel therapeutic indication for dupilumab. This particular case is unique in two aspects when compared to previously reported case series and metanalyses that have examined over 100 Wells syndrome cases.^1^, ^2^, ^3^, ^4^ First, Wells syndrome is most commonly reported on the extremities, trunk, and the face.^1^, ^2^, ^3^, ^4^ To our knowledge, this is the first report that describes a primary lesion localised to the vertex scalp. Therefore Wells syndrome should be considered in the differential diagnosis of localised recalcitrant inflammatory skin lesions irrespective of their anatomic location. Second, the histopathologic criteria of Wells syndrome typically involves a predominant infiltration of reactive eosinophils and histiocytes within the affected dermis.^1^, ^2^, ^3^, ^4^ In addition to these classic features, this case also demonstrated the presence of focally dense neutrophils and highlights this rare neutrophilic subtype of Wells syndrome. Research over the last decade demonstrated the involvement of neutrophils in the establishment of a type 2 response, as dsDNA associated with neutrophils may directly contribute to the pathogenesis by inducing a type 2 immune response.^5^ Therefore it is possible that neutrophils might also play a role in subtype of Wells syndrome possibly associated with a more severe and harder to treat subtype as shown in neutrophil‐rich asthma and chronic rhinitis.^5^ We are hoping our case will open up discussion on the possible role of neutrophil involvement in eosinophilic dermatosis. Further research is necessary for exploring and elucidating the underlying mechanism of neutrophil and eosinophil interplay in Type II responses.

Wells syndrome could be difficult to diagnose due to shared clinical features with other eosinophilic rich inflammatory disorders and lack of definite diagnostic test. Differential diagnosis includes bacterial and fungal infection, Churg‐Strauss syndrome, bullous pemphigoid and parasite infection, etc. Therefore additional work‐up including laboratory test, tissue culture, and imaging are necessary to rule out other possible diagnosis.

The overall recalcitrant nature of this lesion led to the innovative use of dupilumab combined with oral corticosteroids which resulted in complete resolution. This particular treatment regimen proved to be clinically efficacious similar to a case recently described by Traidl et al.^6^ One distinction between our case and that described by Traidl et al was that their patient had an additional history of severe eosinophilic asthma and nasal polyps and was previously being treated with benralizumab (IL‐5 receptor antibody).^6^ Furthermore, the patient interestingly had a relapse of their skin lesions 1 month into initiating dupilumab, however the patient's condition ultimately resolved after 6 months of dupilumab therapy. These shared observations warrant future consideration of the role that IL‐4 and IL‐13 receptor inhibition may play in the treatment of eosinophilic cellulitis, as it is postulated that dupilumab may function by modulating eosinophil trafficking to the dermis or by inhibiting the release of IL‐5 through an IL‐4 mediated mechanism within eosinophils.^6^, ^7^ In conclusion, for refractory cases of Wells syndrome that are not responsive to first‐line therapies, dupilumab may function as a potential treatment option.

## CONFLICTS OF INTEREST

None to declare.

## AUTHOR CONTRIBUTIONS

**Patrick McMullan**: writing – original draft (equal). **Kristin Torre**: conceptualisation (equal), data curation (equal). **Sueheidi Santiago**: conceptualisation (equal), data curation (equal). **Gillian Weston**: data curation (equal). **Jun Lu**: conceptualisation (equal), data curation (equal), supervision (equal), validation (equal), writing – review & editing (lead).

## ETHICS STATEMENT

Not applicable.

## Data Availability

Data sharing not applicable to this article as no datasets were generated or analysed during the current study.
